# Case Report: Squamous Cell Carcinoma of Pancreas With High PD-L1 Expression: A Rare Presentation

**DOI:** 10.3389/fonc.2021.680398

**Published:** 2021-07-01

**Authors:** Baohong Yang, Haipeng Ren, Guohua Yu

**Affiliations:** Oncology Department, Weifang People’s Hospital, Weifang Medical University, Weifang, China

**Keywords:** pancreas cancer, immunotherapy, chemotherapy, squamous cell carcinoma, anti-PD 1

## Abstract

Primary pancreatic squamous cell carcinoma is sporadic. The diagnosis is usually made following surgery or needle biopsy and requires a thorough workup to exclude metastatic squamous cell carcinoma. Squamous cell carcinoma of the pancreas often has a very poor prognosis. There is no treatment guideline for this type of cancer, and to date, no therapeutic regimen has been proven effective. Here, we report the effectiveness of immunotherapy in combination with chemotherapy against locally advanced squamous cell carcinoma of the pancreas with high programmed cell death ligand 1 (PD-L1) expression. Regional intra-arterial infusion chemotherapy consisting of nab-Paclitaxel followed by gemcitabine infused *via* gastroduodenal artery every three weeks for two cycles. This therapy resulted in the depletion of carcinoma, and the patient continues to lead a high-quality life with no symptoms for more than 16 months.

## Study Highlight

1. Regional intra-arterial infusion chemotherapy and partial splenic embolization is administered along with Immunotherapy targeting PD-L1 with gemcitabine to achieve partial remission of a primary locally advanced squamous cell carcinoma of the pancreas with a high level of PD-L1 expression.
2. The patient continues to lead a high-quality life with no symptoms for more than 16 months.
3. PD-L1 may be a favorable prognostic factor for the pancreas’ primary squamous cell carcinoma; blocking the PD-1/PD-L1 pathway combined with chemotherapy would be a rational treatment for pancreatic squamous cell carcinoma patients.

## Case Report

A 54-year-old Chinese woman was referred to our hospital with complaints of fatigue and upper abdominal pain for more than three months. She had a progressive weight loss of 7 kg. In May 2019, she had an abdominal CT scan to evaluate chronic epigastric pain; a solid mass of the pancreas’ neck was detected, measuring 5cm in the maximum diameter. Upper digestive endoscopy showed signs of chronic gastritis and no esophageal lesions. She has no smoking or drinking habits. Her mother had died from pancreatic cancer at the age of 69.

**Figure 1:**
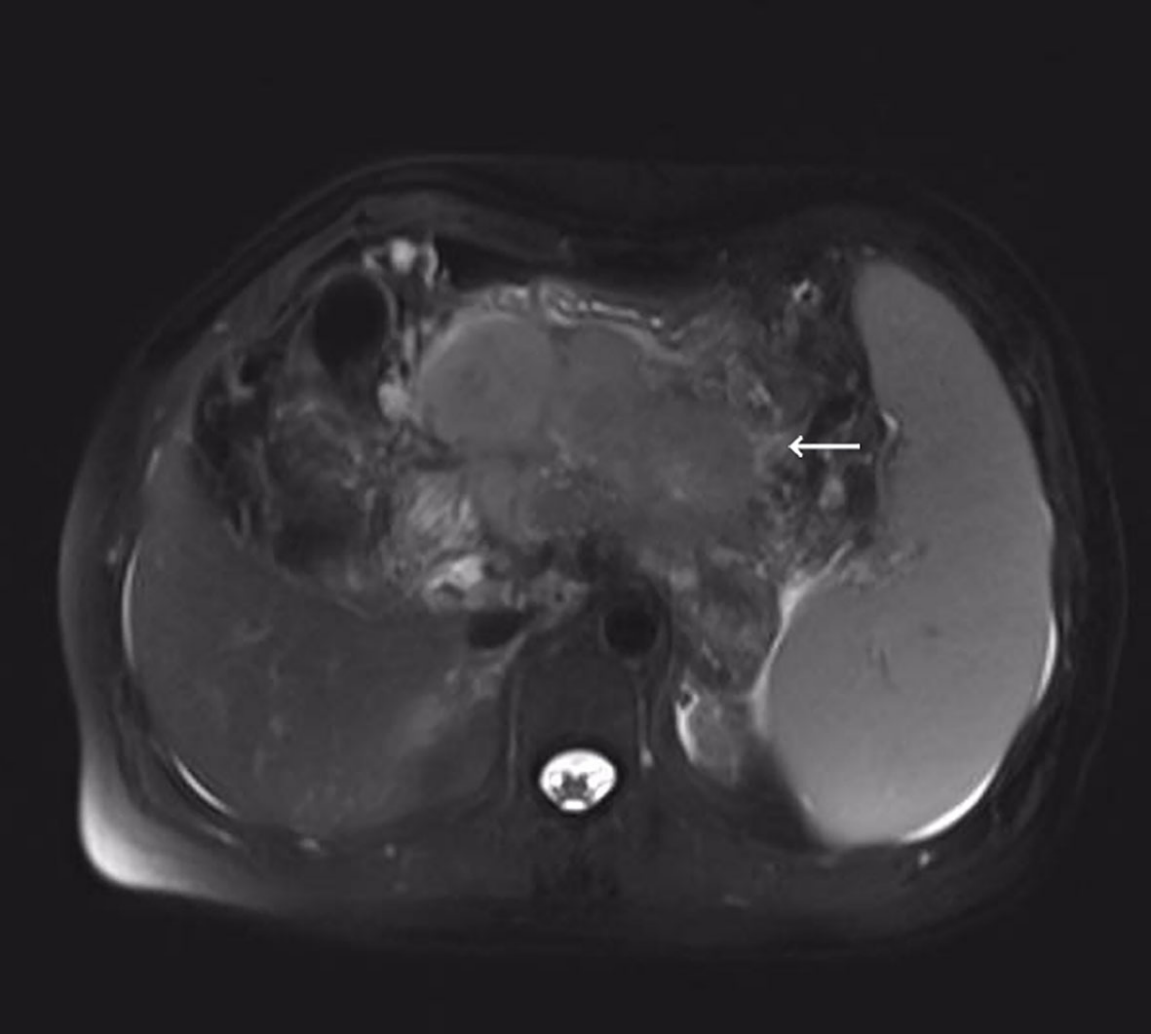
T2-weighted magnetic resonance imaging (MRI) showed irregular pancreatic mass measuring 105 mm in the maximum diameter.

Cancer serology done at our department showed elevated serum levels of squamous cell carcinoma antigen. The carbohydrate antigen 19-9, alpha-fetoprotein, and carcinoembryonic antigen were within the normal range. The serous level of aspartate aminotransferase (AST) was 29 U/L (reference, 8-42), alanine aminotransferase (ALT) 14 U/L (reference, 5-40), alkaline phosphatase (ALP) 72U/L (reference, 34-150), and glutamyl transpeptidase (GGT) 47 U/L (reference, 3-64). A 10.5 cm long hypodense mass with marginal enhancement in the pancreas’ neck was observed on enhanced computed tomography (CT). The T2-weighted magnetic resonance imaging (MRI) showed varying degrees of low-intensity lesions outside the tumor in the pancreatic neck and heterogeneous high-intensity lesions within the tumor (**Figure 1**). Diffusion-emphasized MRI showed high-intensity lesions and involved the posterior potion of the upper gastric body, the splenic vein, and the second segment of the duodenum. MRI and CT also showed splenomegaly and varying degrees of biliary and pancreatic duct dilatation. According to the American Joint Committee on Cancer (AJCC) TNM classification (8th edition), the tumor stage was T4N1M0 (stage III).

**Figure 2:**
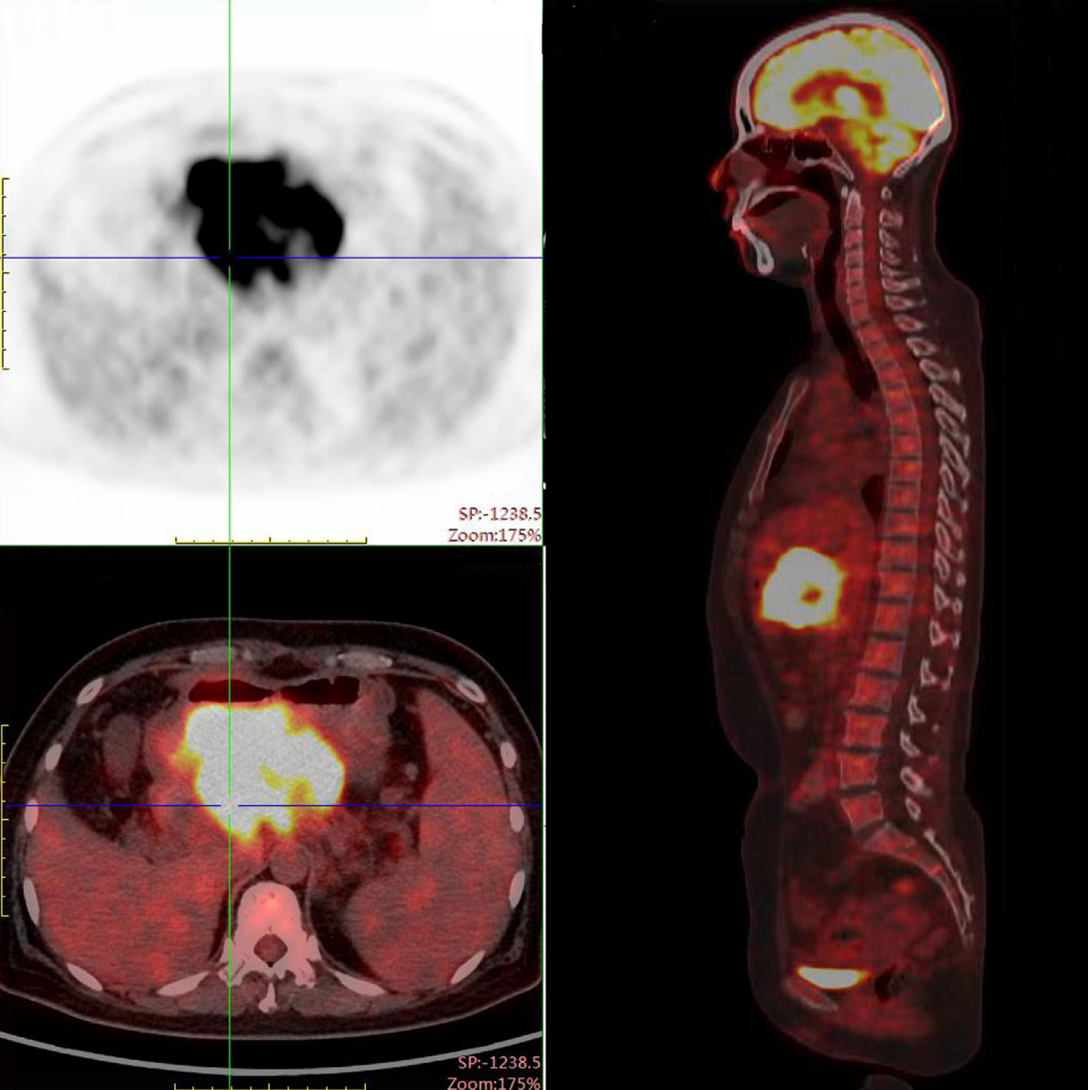
PET-CT scan demonstrated a mass measuring 105 x100 x 70 mm with peripheral elevated FDG avidity in the neck of the pancreas.

**Figure 3:**
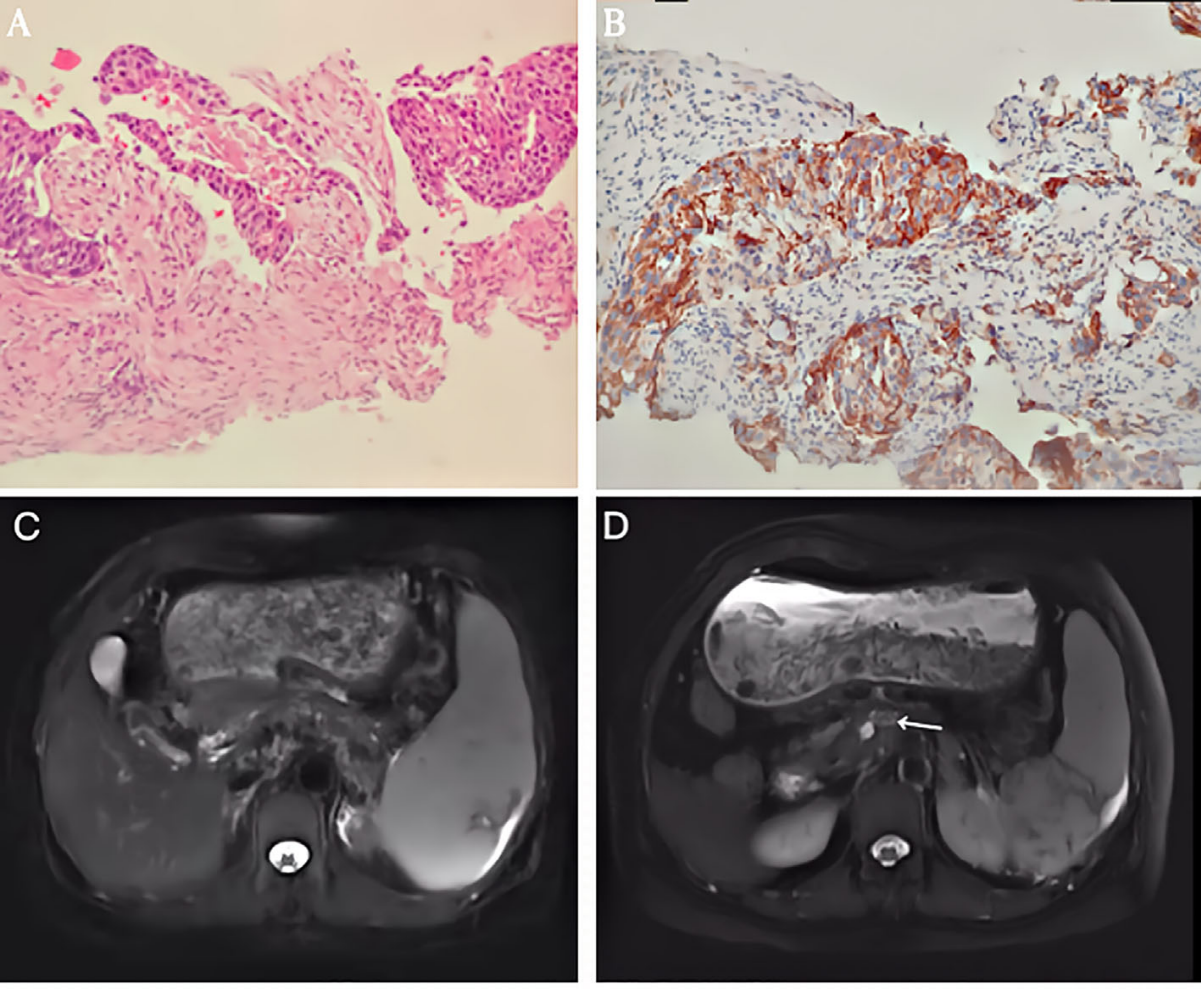
**(A)** Histological findings revealed a pancreatic squamous cell carcinoma (SCC) with abundant eosinophilic cytoplasm and large vesicular nucleus. (HE x 200). **(B)** Immunohistochemical staining of PD-L1 expression. Original magnification, x200. **(C)** T2-weighted magnetic resonance imaging (MRI) showed the mass decreased to 55 mm in the maximum diameter. **(D)** T2-weighted magnetic resonance imaging (MRI) showed the mass measured 16mm after 2 cycles of intra-arterial chemotherapy.

**Figure 4:**
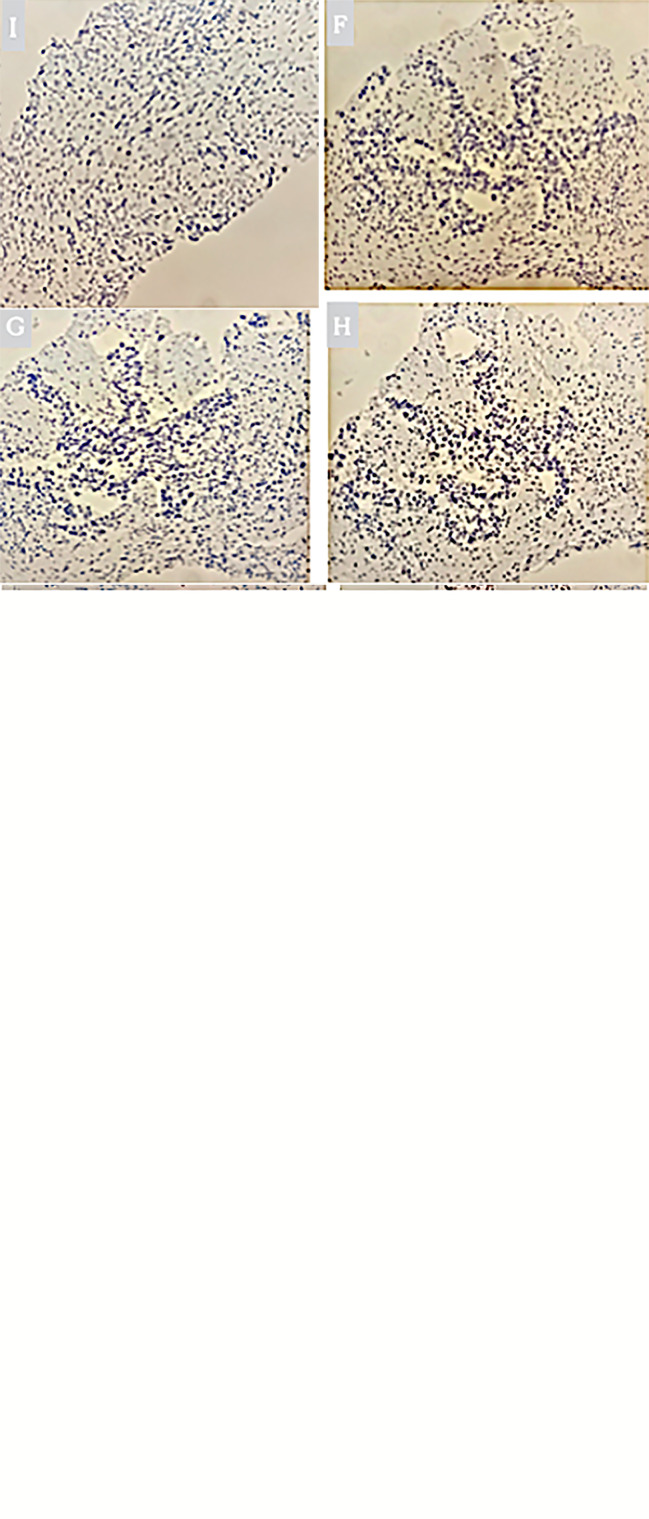
**(A)** Immunohistochemical staining of CK5/6 expression. Original magnification, x200. **(B)** Immunohistochemical staining of CK19 expression. Original magnification, x200. **(C)** Immunohistochemical staining of p63 expression. Original magnification, x200. **(D)** Immunohistochemical staining of GATA-3 expression. Original magnification, x200. **(E)** Immunohistochemical staining of negative CK20 expression. Original magnification, x200. **(F)** Immunohistochemical staining of negative CDX2 expression. Original magnification, x200. **(G)** Immunohistochemical staining of negative Syn expression. Original magnification, x200. **(H)** Immunohistochemical staining of negative Cg A expression. Original magnification, x200. **(I)** Immunohistochemical staining of negative TTF-1 expression. Original magnification, x200.

PET/CT examination of f-fluorodeoxyglucose (F-FDG) showed high coalescence of FDG at the site of the pancreatic neck mass with a maximum standardized uptake value (SUV max) of 26.7, while the SUV max of the surrounding lymph nodes was 30.6 (**Figure 2**). A percutaneous biopsy of the pancreatic tumor was performed under CT guidance, and pathological histology shows squamous cell carcinoma with distinct keratinized features, abundant eosinophilic cytoplasm, and large vesicular nuclei (**Figure 3A**). Immunohistochemical analysis revealed that the malignant cells were positive expression of CK5/6, CK19, p63, and GATA-3. TTF-1, CK20, CDX2, CgA, and Syn were negative (**Figures 4A–I**). Expression of the PD-L1 protein was evaluated with an immunohistochemical method using a mouse monoclonal anti-human PD-L1 antibody (Clone 22C3, Dako). PD-L1 positive tumor cells within the tumor tissue specimen was 90% (**Figure 3B**), the expression of MSH2, MLH1, MSH6, and PMS2 were positive.

Considering her poor physical condition and the presence of peripheral tissue and vascular involvement. We first administered immunotherapy inhibiting the programmed cell death protein 1 (PD 1)/PD-L1 pathway using Sintilimab injection (Tyvyt^®^) (200mg, q21d) plus chemotherapy using gemcitabine (1000mg/m^2^, d1,8, q21d). After completing two courses of the combined therapy, abdominal pain was relieved. The abdominal CT showed the pancreatic mass had shrunk to 7 cm, achieving a partial response. Grade 3 thrombocytopenia appears after eight cycles of combined therapy. Then, Sindilimab monotherapy was administered every three weeks, and repeated MRI imaging showed the mass had shrunk to 5.5 cm after seven cycles of single immunotherapy (**Figure 3C**). To improve the efficiency, we administered regional gastroduodenal arterial infusion chemotherapy with nab-Paclitaxel at a dose of 125mg/m^2^, followed by gemcitabine (1000mg/m^2^). This patient received two cycles of intra-arterial infusion in three weeks. Meanwhile, partial splenic embolization using one vial of 100–300 microspheres (Bead Block^®^ Terumo) was done because of grade 3 thrombocytopenia. No drug-related adverse events were noted after treatment. The platelets gradually returned to normal. MRI of the upper abdomen showed that the tumor regressed to 1.6 cm 1 month after two intra-arterial infusion therapy cycles (**Figure 3D**). She was followed up for an additional four months with no relapse and lives a high quality of life more than 16 months after the initial treatment.

## Discussion

Among all primary pancreatic exocrine malignancies, pancreatic ductal squamous cell carcinoma accounts for about 0.5% ~ 5% (1). Although the origin of pancreatic SCC is still unclear, observations indicate that it may originate from adenocarcinomas with squamous metaplasia, where the tumor may drift into adenocarcinoma or SCC. The diagnosis of pancreatic SCC is complicated, requiring a thorough investigation to rule out related carcinomas (2). Up to now, no promising treatment options are available. Gemcitabine has been considered the standard chemotherapy option for pancreatic ductal adenocarcinoma since 1997, but there is limited data on its use in SCC. Kataoka et al. demonstrated overall survival (OS) of 7 months after treatment with nab-paclitaxel and gemcitabine in a patient with pancreatic adenosquamous carcinoma (3). Anagnostopoulos et al. found gemcitabine-based chemoradiotherapy was effective in the case of pancreatic SCC (4). Recently, Ntanasis-Stathopoulos et al. reported a systematic analysis showing a median overall survival (OS) of 7 months in 54 patients with primary pancreatic squamous cell carcinoma. In the subgroup analysis, the median overall survival was ten months in resectable cases and only four months in unresectable cases (5). Since there has been little change in the survival outcomes of pancreatic SCC for decades, it has been necessary to develop new therapies.

Recently, immune checkpoint inhibitors (ICI) inhibiting PD 1/PD-L1 pathway have shown remarkable therapeutic efficacy in many types of cancer. Combined checkpoint inhibitor use has been approved by the American Food and Drug Administration (FDA) and combination with chemotherapy. The expression of PD-L1 is extremely low in pancreatic ductal adenocarcinoma (PDCA) relative to the observed frequencies of about 30-40% in colon and stomach cancers (6–9). Interestingly, Masahiko et al. observed high expression of PD-L1 exclusively in the SCC component of pancreatic adenosquamous carcinoma samples. Moreover, mismatch repair gene (MMR) associated proteins were all positively expressed in both the squamous and adenocarcinoma components (10). In this case, expression levels of PD-L1 in pancreatic squamous cell carcinoma were 90%, but there was no loss of function of MMR proteins in the tumor cells.

Drugs targeting the PD-1/PD-L1 pathway may have an improved therapeutic effect on tumors expressing higher levels of PD-L1 (11). Anti-PD-1 inhibitors may be a sensible option to treat pancreatic malignant tumors with high expression of the PD-L1 protein. In 2007, Takeo et al. first reported that PD-L1 is a new prognostic marker and the PD-L1/PD-1 signalling pathway may play a critical role in the development of pancreatic cancer. Anti-PD-L1 monoclonal antibody combined with gemcitabine showed a significant synergistic anti-tumor effect with complete tumor regression in a mouse model (12).

Sintilimab Injection (Tyvyt^®^) is a fully human immunoglobulin G4 monoclonal antibody that blocks the PD-L1 signalling pathway by binding to PD-1 molecules on the surface of T lymphocytes and triggers T lymphocytes to attack cancer cells. Sintilimab injection is approved for relapsed or refractory classical Hodgkin’s lymphoma in China. Phase I/II clinical studies of Sintilimab in solid tumors are ongoing in the U.S. Like other anti-PD-1 drugs, it may cause side effects in different parts of the body, which may occur during treatment or several months after treatment is completed. In this case, the immune-related adverse event occurred after two cycles of therapy. Grade 1 pruritis occurred within 72 hours of Sintilimab infusion and resolved with topical emollients and mild topical corticosteroids; the symptom did not worsen with subsequent infusions. Overall, treatment was well tolerated and no severe adverse reactions (SAEs) were observed.

Immunotherapy targeting PD-1 with a gemcitabine regimen is the first time used to treat primary advanced squamous cell carcinoma of the pancreatic neck with high-level expression of the PD-L1 protein. We have also achieved a partial remission of the neoplasm in this patient, suggesting that PD-L1 expression may be a favorable prognostic factor for the pancreas primary squamous cell carcinoma and inhibits the PD-1/PD-L1 pathway combined with chemotherapy may be helpful in treatment for pancreatic SCC patients.

## Data Availability Statement

The raw data supporting the conclusions of this article will be made available by the authors, without undue reservation.

## Ethics Statement

The studies involving human participants were reviewed and approved by Weifang People’s Hospital. The patients/participants provided their written informed consent to participate in this study.

## Author Contributions

BY drafted the manuscript. HR and GY revised the manuscript. All authors contributed to the article and approved the submitted version.

## Conflict of Interest

The authors declare that the research was conducted in the absence of any commercial or financial relationships that could be construed as a potential conflict of interest.

## Abbreviations

SCC, squamous cell carcinoma; F-FDG, F-fluorodeoxyglucose; SUV max, maximum standardized uptake value; MMR, mismatch repair gene; PDAC, pancreatic ductal adenocarcinomas; FDA, Food and Drug Administration; ICIs, Immune checkpoint inhibitors; CTLA-4, cytotoxic T-lymphocyte-associated antigen-4; PD‐1, programmed cell death protein 1; OS, overall survival.
